# Epidemiological characterization of chronic myeloid leukaemia patients at an oncologic centre: A retrospective observational study

**DOI:** 10.1016/j.htct.2025.103935

**Published:** 2025-07-05

**Authors:** Ana Maria Meireles, Rita Calisto, Maria José Bento, Pedro Martinho Gouveia, Susana Bizarro, Manuel Teixeira, Cláudia Moreira, Ana Espírito Santo, Mário Mariz

**Affiliations:** a Department of Hematology and Bone Marrow Transplantation, Instituto Português de Oncologia do Porto (IPO Porto), Porto, Portugal; b Clinical Oncology Group, IPO Porto Research Center (CI-IPOP), Instituto Português de Oncologia do Porto (IPO Porto), Porto, Portugal; c Group of Epidemiology, Results, Economy and Management in Oncology – Research Center, Porto Comprehensive Cancer Center (Porto. CCC) & RISE@CI-IPOP (Health Research Network), Porto, Portugal; d Population Studies Department. School of Medicine and Biomedical Sciences, ICBAS, University of Porto, Portugal Epidemiology Service, Instituto Português de Oncologia do Porto (IPO Porto), Porto, Portugal; e Unidade Local de Saúde Santo António, Porto, Portugal; f Oncogenetics Group, IPO Porto Research Center (CI-IPOP), Instituto Português de Oncologia do Porto (IPO Porto), Porto, Portugal; g Instituto Português de Oncologia do Porto (IPO Porto), Porto, Portugal

**Keywords:** Chronic myelogenous leukaemia BCR-ABL positive, Imatinib mesylate, Tyrosine kinase inhibitors, Epidemiology, Myeloproliferative disorders

## Abstract

**Background:**

The chronic myeloid leukaemia population, treatment patterns and responses in Portugal are unknown. The aim of this study is to describe these features in a Portuguese reference centre.

**Methods:**

A retrospective cohort study included patients with chronic myeloid leukaemia, treated between 2012 and 2022 at the Instituto Português de Oncologia of Porto. Data were obtained from the Cancer Registry of the institution and clinical records. Variables included demographic data, treatments administered, responses (hematologic, cytogenetic, major and deep molecular responses), adverse events, and survival. Patients without available data, those treated in a clinical trial context, and those admitted only for hematopoietic transplantation were excluded.

**Results:**

Ninety-nine patients were included in this study, with a median age of 52 years (range: 7–84 years) at diagnosis. The first-line treatment was imatinib in 96 patients however 33 required second-line with dasatinib, and 17 discontinued treatment while maintaining response. Regarding responses, 95 (96 %) patients achieved cytogenetic response, 90 (94 %) achieved major molecular response, and 71 (72 %) achieved deep molecular response. At three months, the early molecular response rate was 77 %. At 12 months of treatment, of the 67 patients with response evaluation, 93 % achieved complete cytogenetic response and 49 % major molecular response. Both imatinib and dasatinib were well tolerated. The median follow-up was eight years. The five-year overall survival was 96 %.

**Conclusion:**

This study is the first to characterize chronic myeloid leukaemia patients at a Portuguese centre. The patient characteristics, responses, and overall survival were within the expected range according to the literature. This study confirms the good prognosis of chronic myeloid leukaemia and the good responses using imatinib as first-line treatment.

## Introduction

Chronic myeloid leukaemia (CML) is a myeloproliferative neoplasm that accounts for 15 % of adult leukaemias and whose prevalence has been increasing.^1^ This pathology is defined by the presence of a *BCR::ABL1* fusion gene, which encodes a protein with the same name. The expression of this fusion protein leads to the activation of signaling pathways. This activation contributes to increased cell division, reduced apoptosis, and altered adhesion to stromal cells/extracellular matrix, leading to the leukaemic phenotype.^2^^,^^3^

CML is more common in older individuals.^1^ The natural history of the disease includes three phases: a chronic phase, an accelerated phase, and a blastic phase.^4^ Until 2000, only 10 % of patients achieved a complete cytogenetic response.^5^ However, with the study of the disease's pathophysiology and the subsequent development of tyrosine kinase inhibitors (TKIs), the paradigm and course of this disease have changed substantially.^6^

Currently, CML has become a chronic disease, requiring regular molecular monitoring, adherence to TKI therapy, and proper management of toxicities.^7^ Patients adequately treated with these agents, who have a good response to TKI, have a survival rate similar to the general population.^8^, ^9^, ^10^, ^11^, ^12^ Moreover, some patients may discontinue TKI therapy and maintain remission. Since the approval of imatinib in 2001, the therapeutic arsenal for CML has expanded with the introduction of other TKIs such as nilotinib, dasatinib, bosutinib, ponatinib, and asciminib.^8^, ^9^, ^10^, ^11^^,^^13^^,^^14^ However, clinical trial candidates are not the same as real-world patients, and several authors argue for the need to understand these outcomes.^15^^,^^16^ Although many countries have epidemiological databases and published literature on real-world outcomes for their CML patients,^17^, ^18^, ^19^, ^20^, ^21^ to our knowledge, there are no published studies on these outcomes in Portugal.

The aim of this article is to describe the population of CML patients treated at Instituto Português de Oncologia do Porto (IPO-Porto), including the characterization of the patients, treatment patterns, therapeutic efficacy, documented adverse effects of TKIs, number of candidates for TKI discontinuation, and whether there was a need to resume TKI therapy.

## Materials and methods

This retrospective cohort, single-centre study was conducted at the Haematology and Bone Marrow Transplantation Department of IPO-Porto. Patients were selected from the Cancer Registry of the institution. The inclusion criteria were: patients with CML (International Classification of Diseases for Oncology, 3rd Edition, ICD-O-3: 9863/3; 9875/3; 9876/3) treated at IPO-Porto between 1/11/2012 and 31/10/2022 and aged 18 years or older during the study period. Patients whose data were unavailable, incorrect diagnoses, patients admitted solely for hematopoietic progenitor cell transplantation, or those treated in a clinical trial context were excluded. The follow-up of patients continued until death, loss to follow-up, or administrative closure of this project in May 2024.

Patient data were collected through electronic medical records and cancer registry. The extracted variables included:

1. At the time of diagnosis: gender, age, Charlson comorbidity index,^22^ clinical presentation (number of leukocytes, percentage of peripheral blasts, splenomegaly, constitutional symptoms, disease phase), presence of additional cytogenetic abnormalities, type and quantity of *BCR::ABL1* transcript, and prognostic scores: Sokal Index for CML (SOKAL),^23^ and EUTOS long-term survival (ELTS) score.^24^
2. During treatment: the treatments administered, hematological, cytogenetic, major and deep molecular responses, adverse events, the need for TKI switch and reasons for the switch, progression, death, cause and date of death were collected.

Response evaluation was conducted according to the European Leukaemia Net 2020 recommendations.^6^ Progression was defined as death from any cause, loss of cytogenetic response, or progression to accelerated or blastic phase.

For event-free survival (EFS), an event was defined as the first occurrence of any of the following: death from any cause during treatment, progression to accelerated-phase CML (characterized by ≥15 % blasts in the blood or bone marrow, ≥30 % blasts plus promyelocytes in the blood or bone marrow, ≥20 % peripheral basophils, or thrombocytopenia <100 × 10^3^/µL unrelated to treatment), or progression to blast-phase CML (defined by ≥30 % blasts in the blood or bone marrow or extramedullary involvement, excluding hepatosplenomegaly). Loss of complete hematologic response (CHR) was defined by the occurrence of any of the following in two blood samples obtained at least one month apart: a white blood cell count >20 × 10^3^/µL, a platelet count ≥600 × 10^3^/µL, the appearance of extramedullary disease, ≥5 % myelocytes and metamyelocytes in the peripheral blood, or the presence of blasts or promyelocytes in the peripheral blood. Loss of major cytogenetic response (MCyR) was defined as an increase in Philadelphia chromosome-positive (pH^+^) cells in metaphase by at least 30 percentage points on two cytogenetic analyses performed at least one month apart. An increasing white blood cell count was defined as a doubling to >20 × 10^3^/µL measured on two occasions at least one month apart in a patient who had never achieved a strict CHR despite receiving maximally tolerated doses of therapy. Overall survival (OS) was calculated from time to death from any cause. The criteria for discontinuing TKI therapy was based on the 2020 European Leukaemia Net guidelines.^6^

### Statistical analysis

For descriptive analysis, categorical variables were presented as frequencies and percentages, and continuous variables as medians and ranges. Survival analysis was performed using the Kaplan-Meier estimator. Statistical analysis was conducted using the software R.

### Ethical consideration

This project was submitted to the Ethics Committee of IPO-Porto (Ref. CES. 79/023). All data were processed in accordance with European and Portuguese data protection laws.

## Results

### Patient characteristics

Data from 157 patients were extracted from the oncologic registry of the IPO Porto. Of these, 58 cases were excluded for the following reasons: a diagnosis other than CML, lack of follow-up at the institution, referral solely for hematopoietic stem cell transplantation, inclusion in clinical trials, or unavailable data.

The median age at diagnosis of the 99 patients included was 52 years (range: 7–84 years), with a slight predominance of males (57 %). Two of the included patients were under 18 at the time of diagnosis, but were followed up only after becoming 18 years of age. The median Charlson comorbidity index was 0 (range: 0–9), and hypertension was the most common comorbidity (40 %). The patient characteristics are detailed in Table 1. Most patients were in the chronic phase at diagnosis (*n* = 84); however, for 15 patients, the disease phase could not be determined. The total of high-risk patients was 14 (14 %) and 5 (5 %) according to the SOKAL and the ELTS scores, respectively. The transcript was classic (e13a2 or e14a2) in 74.8 % of patients. The disease characteristics are described in Table 2.

**Table 1:** Characteristics of the study population.

	Overall
	(*n* = 99)
Gender – *n* (%)	
Female	43 (43.4)
Male	56 (56.6)
Age at diagnosis^a^	
Median (range)	52.0 (7.00–84.0)
ECOG – *n* (%)	
0	84 (84.8)
1	10 (10.1)
2	3 (3.0)
3	1 (1.0)
4	1 (1.0)
Charlson comorbidity index (excluding the presence of CML +2)	
Median (range)	1.00 (0–9.00)
Smoker - *n* (%)	
No	87 (87.9)
Yes	12 (12.1)
Hypertension - *n* (%)	
No	59 (59.6)
Yes	40 (40.4)
Dyslipidaemia - *n* (%)	
No	70 (70.7)
Yes	29 (29.3)
Diabetes - *n* (%)	
No	84 (84.8)
Yes	15 (15.2)
Previous cardiovascular event - *n* (%)	
No	93 (93.9)
Yes	6 (6.1)

a Although two patients were under 18 years of age at the time of diagnosis, their follow-up in this study only began once they reached 18.

**Table 2:** Baseline chronic myeloid leukaemia characteristics.

	Overall
	(*n* = 99)
Splenomegaly - *n* (%)	
No	57 (57.6)
Yes	22 (22.2)
Unknown	20 (20.2)
Spleen size (mm below the costal margin)	
Median (range)	0 (0–100)
Missing - *n* (%)	21 (21.2)
Constitutional symptoms - *n* (%)	
No	60 (60.6)
Yes	23 (23.2)
Unknown	16 (16.2)
Disease stage - *n* (%)	
Chronic	84 (84.8)
Unknown	15 (15.2)
SOKAL Score - *n* (%)	
High	14 (14.1)
Intermediate	32 (32.3)
Low	33 (33.3)
Unknown^b^	21 (21.2)
ELTS Score - *n* (%)	
High	5 (5.1)
Intermediate	26 (26.3)
Low	47 (47.5)
Unknown	21 (21.2)
Additional cytogenetic abnormalities at diagnosis - *n* ( %)	
No	66 (64.6)
Yes^a^	4 (4.0)
Unknown^b^	29 (29.3)
Transcript type - *n* ( %)	
e13a2	36 (36.4)
e14a2	33 (33.3)
e13a2 and/or e14a2	5 (5.1)
e1a2	3 (3.0)
e19a2	1 (1.0)
Not found	1 (1.0)
Missing	20 (20.2)

a 3 patients had a second pH, and the other clonal evolution −1.

b Unavailable data.

### Treatment

First-line treatment was imatinib 400 mg (Glivec®) in 96 patients (97 %). Other treatments included nilotinib (*n* = 1), interferon (*n* = 1) and interferon with hydroxyurea (*n* = 1). The median time from diagnosis to initiation of treatment was 13 days (range: 0–45 days).

Among patients treated with imatinib, 32 required dose adjustments. Three patients escalated to imatinib 600 mg due to insufficient response. An insufficient response was defined as failure to achieve *BCR::ABL1* <1 % (International Scale) at 12 months of TKI therapy. Of the 29 patients who reduced their dose, 13 did so due to toxicity, initially decreasing to 300 mg and later to 200 mg. Sixteen patients reduced their dose due to sustained deep molecular responses (≥4 years) before attempting TKI discontinuation. Additionally, 34 (35 %) patients switched TKIs: 30 due to insufficient response and four due to intolerance. Criteria for discontinuing imatinib according the ELN guidelines of 2020^6^ was met in 22 patients: 11 maintained molecular responses with TKI suspension and 11 needed to resume treatment, responding quickly to the reintroduction.

Second-line treatment was dasatinib in 33 patients. Other treatments included azacitidine associated with interferon and imatinib. Of these, 11 changed the dose: two increased due to insufficient response, and the remaining nine reduced the dose. Criteria for discontinuing dasatinib was met in one patient, who maintained sustained molecular response.

**Figure 1:**
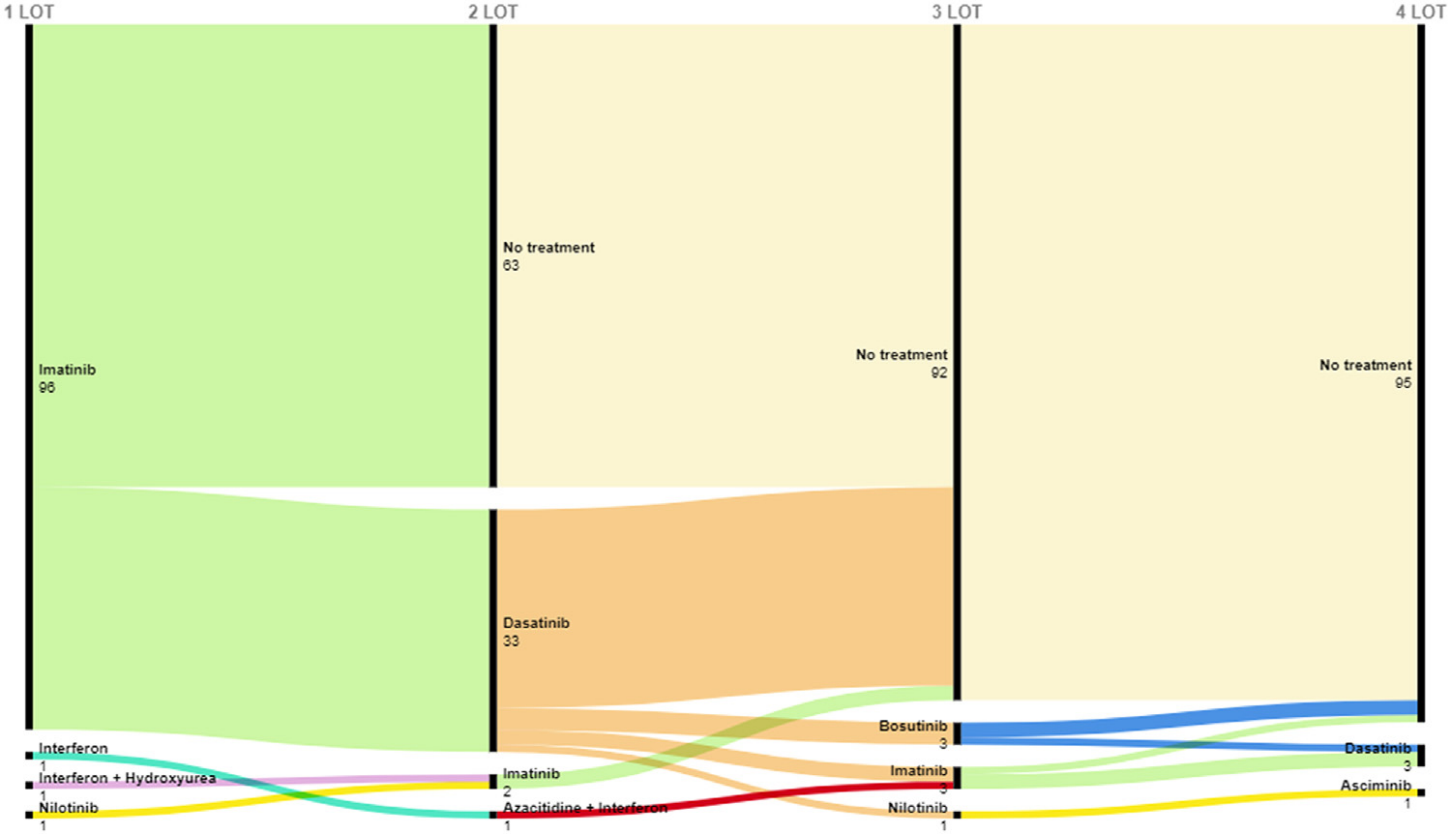
Treatment patterns during the study period.

There was no preferential treatment for 3rd and 4th lines. The treatments administered and their sequence are illustrated in Figure 1. Only two patients were treated with allogenic stem cell transplantation.

### TKI domain mutation testing

All the patients who had treatment failure (*n* = 31) underwent TKI domain mutation testing, confirming mutations in eight patients: one mutation conferred partial resistance to imatinib (p.(Cys475*)); five conferred complete resistance (Q252R, M388L, M244V, F311L, G250R); one patient had two mutations conferring resistance to imatinib and nilotinib simultaneously (E255V and Y253H), as is described in Table 3. The remaining mutations had indetermined significance, including c.708G>*T*, p.(Glu236Asp): and N322S. The majority of these mutations are not mentioned in reports as they are not significant.

**Table 3:** Identified mutations which configured resistance to tyrosine kinase inhibitors.

Mutation	Resistance
Q252R	Resistance to Imatinib
Y253H	Resistance to Imatinib and Nilotinib
G250R	Insensitivity to Imatinib
p.(Cys475Tyrfs*11) in ABL1	Resistance to Imatinib
F311L	Insensitivity to Imatinib
p.(Cys475*)	Of controversial significance, reported as conferring partial resistance to Imatinib
M244V	Resistance to Imatinib
M388L	Insensitivity to Imatinib

### Adverse effects

During the median follow-up of 8.25 years (Interquartile Range [IQR]: 7.14 years), the most common adverse events of imatinib including all lines of treatment (*n* = 101 – two patients who restarted imatinib at a later point were counted as having an additional line of therapy) were: gastrointestinal (39 %), myalgias (23 %), oedema (19 %), hematologic changes (13 %), fatigue (13 %), arthralgias (11 %), and rash (6 %). For dasatinib (*n* = 36), the most notable effects were: pleural effusion (25 %) and hematologic changes (8 %). For bosutinib (*n* = 3), one patient experienced cardiac toxicity and another gastrointestinal toxicity. For nilotinib (*n* = 2), one patient experienced critical limb ischemia. Only one patient was treated with asciminib, experiencing hypertension.

The adverse events reported prompted a change of TKI due to intolerance in 4 % of patients treated with imatinib, 11 % with dasatinib (pleural effusion), and 50 % of patients treated with nilotinib. Grade 3 and 4 adverse events by drug are shown in Table 4.

**Table 4:** Grade 3 and 4 toxicities documented during the study.

	Imatinib (*n* = 103)	Dasatinib (*n* = 33)	Bosutinib (*n* = 3)	Nilotinib (*n* = 4)	Asciminib (*n* = 1)
Myalgias	1 (1 %)	0 (0 %)	0 (0 %)	0 (0 %)	0 (0 %)
Pancreatitis	1 (1 %)	0 (0 %)	0 (0 %)	0 (0 %)	0 (0 %)
Rash	2 (2 %)	0 (0 %)	0 (0 %)	0 (0 %)	0 (0 %)
Hematologic toxicity	1 (1 %)	0 (0 %)	0 (0 %)	0 (0 %)	0 (0 %)
Pleural effusion	0 (0 %)	4 (12 %)	0 (0 %)	0 (0 %)	0 (0 %)
Cardiovascular event	0 (0 %)	0 (0 %)	0 (0 %)	1 (25 %)	0 (0 %)
Hepatic toxicity	0 (0 %)	0 (0 %)	0 (0 %)	1 (25 %)	0 (0 %)

### Responses and survival

During the follow-up, 98 patients (99 %) achieved a complete hematologic response, 95 (96 %) achieved a complete cytogenetic response, 90 (94 %) achieved a major molecular response, and 71 (72 %) reached a deep molecular response. At three months, 77 % of patients had achieved an early molecular response. By 12 months, 93 % achieved a complete cytogenetic response, 49 % achieved a major molecular response, and 21 % reached a deep molecular response. Among patients treated exclusively with imatinib, 95.7 % (45/47) achieved a cytogenetic response by 12 months, and 96.7 % (58/60) achieved it during the study follow-up. Among patients with a high risk according to the ELTS score, 75 % achieved a complete cytogenetic response within the first year.

**Figure 2:**
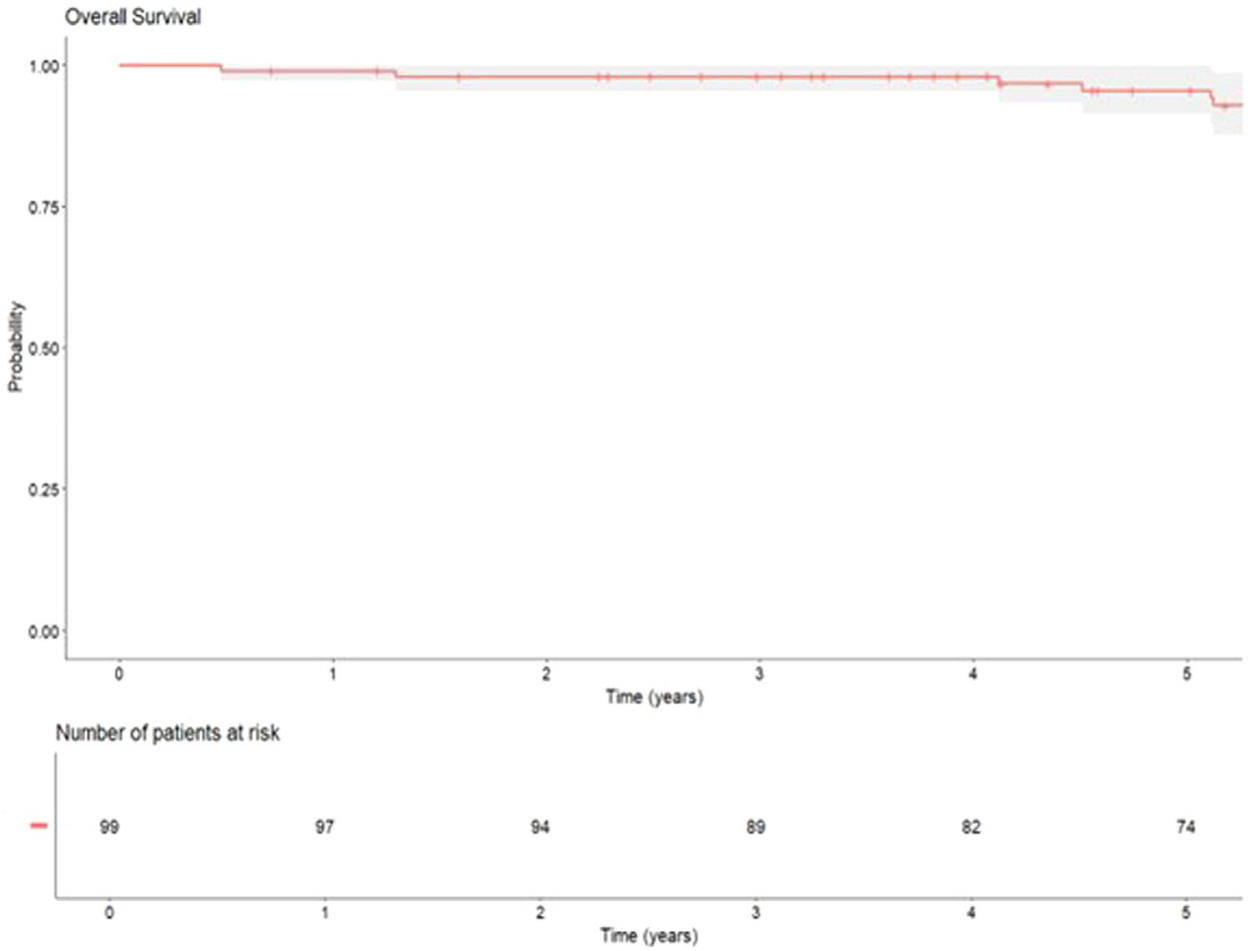
Overall survival of patients with chronic myeloid leukaemia over ten years of follow-up.

During the study period, seven patients lost hematologic or cytogenetic response, though none progressed to the accelerated or blast phase. The median progression free survival was 268 months. The one-year event-free survival rate was 84 %, and at five years, it was 53 %. No patients were excluded due to loss to follow-up. Of under 45-year-old patients (*n* = 19), 47 % achieved a deep molecular response. Overall survival was 88 %, with no deaths due to CML-related causes (Figure 2). The five-year survival rate was 96 %.

## Discussion

This study is, to our knowledge, the first to describe patients with CML in Portugal. The patient population exhibited characteristics similar to those seen in clinical trials, with a similar age (52 years), and small proportion of high-risk patients according to the ELTS score.^8^, ^9^, ^10^, ^11^ Imatinib was the main first-line therapy, with good tolerance. A third of patients needed a second line and a fifth successfully discontinued TKI. The rate of Grade 3 and 4 complications was higher in patients with TKI of higher generations. The responses achieved were comparable to expectations, with a complete cytogenetic response of 93 % and major molecular response at 12 months of 49 %, as was the overall survival.

This study reinforces the good outcomes currently achieved in CML. All TKIs are highly effective in newly diagnosed chronic-phase CML, with long-term overall survival rates comparable to age-matched controls.^25^ This study underlines the results obtained in clinical trials, showing no significant differences in overall survival between patients starting treatment with imatinib versus second-generation TKIs, as described in Table 5.^8^, ^9^, ^10^, ^11^ In this study, the rate of disease progression was low, achieving outcomes as good as those obtained with second-generation TKIs in clinical trials.^9^, ^10^, ^11^ However, the study corroborates the faster achievement of molecular responses and deeper responses with second-generation TKIs, which may facilitate TKI discontinuation in selected patients. At 12 months, 49 % achieved major molecular response, while 94 % achieved molecular response. The good tolerability of TKIs was also confirmed. In this sample, as in the DASISION trial, patients treated with imatinib had more myalgias and peripheral oedema, and those treated with dasatinib had more pleural effusion. Although rare, one patient treated with dasatinib developed pulmonary hypertension.^9^ As demonstrated in the ENESTnd trial, the risk of cardiovascular events was higher in patients treated with nilotinib, even in a small sample (*n* = 4).^11^

**Table 5:** Comparison of the main findings of the present study with other studies in literature.

Variable	Present cohort	Italian cohort^1^	Australian cohort^2^	Brazilian cohort^3^	Spanish cohort^4^
*n*	99	226	86	227	62
Age (median)	53	60	55	50	40
First line treatment	Imatinib 400 mg (97 %)	Imatinib 400 mg (86 %)	Imatinib 400 mg	Imatinib 400 mg (80 %)	Imatinib 400 mg (89 %)
MMR at 12 months (%)	49	55	44	56	91 in all follow-up
Follow up	2012–2022	2008–2012 (followed until 2015)	2001–2018	2007–2017	–
5-year EFS (%)	53 %	93	76	–	–
5-year survival (%)	98	85	94	91	100

CCyR, complete cytogenetic response; MMR, major molecular response; EFS, event free survival.

Several real-world studies have evaluated the efficacy of first-line imatinib treatment in countries such as Italy^17^ and Spain.^18^ These studies showed responses comparable to the present study, with major molecular responses at one year of around 50 %. Other studies evaluated not only first-line imatinib but also second-generation TKIs, including countries like Switzerland,^19^ The Netherlands^20^ and Italy^21^ with heterogeneous treatment patterns and response evaluations. Therefore, comparing study results is difficult.

The selection of first-line treatment remains controversial. Several authors advocate starting treatment with a second-generation TKI^26^; one meta-analysis even recommended the use of second- and third-generation TKIs for younger individuals without comorbidities.^27^ However, the choice of first-line TKI involves considerations not only of age and comorbidities, but also of treatment intent (survival versus TKI discontinuation), risk scores, costs, and availability.^25^^,^^28^^,^^29^

This study shows that first-line imatinib, even for higher-risk and younger patients, continues to be a good option, allowing for excellent responses, good tolerability, and lower financial burden on the National Health Service.

As already mentioned, this study analyses the Portuguese population with CML, including treatment patterns, responses, and adverse effects. Characterizing this population is relevant because it may have different characteristics from other populations and because of the unique organization of the Portuguese healthcare system. The data from this study could potentially inform future therapeutic decisions regarding CML at a national level and improve care for these patients.

This study has limitations, including the small sample size, especially concerning the number of patients treated with second-generation TKIs and inhibitors other than imatinib, which limits the conclusions regarding these drugs. Additionally, due to its retrospective nature, this study is subject to information bias, given that some data were not available.

## Conclusion

This is first study to characterize the Portuguese CML patient. The features, responses, survival, and adverse effects of the population are similar to those described in the literature. Furthermore, this study reinforces the good efficacy-tolerability profile of imatinib as a first-line treatment. A more detailed understanding of the population, treatment patterns, and outcomes in Portugal could improve the clinical practice in the country.

This work was supported by a grant from Associação Portuguesa Contra a Leucemia (APCL).

## Authorship contributions

Ana Maria Meireles contributed to data collection and manuscript writing. Rita Calisto performed the statistical analysis. All other authors—Maria José Bento, Pedro Martinho Gouveia, Susana Bizarro, Manuel Teixeira, Cláudia Moreira, Ana Espírito Santo, and Mário Mariz—contributed to the critical revision of the manuscript and approved the final version for submission.

## Conflicts of interest

The authors declare no conflicts of interest.
